# Health behaviors, outcomes and their relationships among young men aged 18-24 years in a rural area of north India: A cross-sectional study

**DOI:** 10.1371/journal.pone.0220285

**Published:** 2019-07-26

**Authors:** Sumit Malhotra, Shashi Kant, Farhad Ahamed, Ramashankar Rath, Mani Kalaivani, Sanjeev Kumar Gupta, S. Ramadass, Vineet Kumar Pathak, Abhishek Jaiswal, Raghavan Parthasarath, Bhabani Prasad Acharya, Vignesh Dwarakanathan

**Affiliations:** 1 Centre for Community Medicine, All India Institute of Medical Sciences, New Delhi, India; 2 Department of Biostatistics, All India Institute of Medical Sciences, New Delhi, India; University of Sao Paulo Medical School, BRAZIL

## Abstract

**Background:**

There is limited information related to health behaviors and their related factors among young men in rural setting of India. This study was conducted to investigate multiple health risk behaviors and outcomes among young men aged 18–24 years in rural India.

**Methods:**

This was a community-based cross-sectional survey conducted in the Ballabgarh block of Faridabad district, Haryana, India. Information regarding socio-demographic details, substance use, injury & violence, mental health and sexual behaviors were collected using a semi-structured interview schedule. Age adjusted prevalence estimates of behaviors and outcomes are computed along with 95% Confidence Intervals. Mediation analysis was carried out to examine relationships between socio-demographic variables, select behaviors and outcomes reported in the study.

**Results:**

A total of 836 young men participated in the study, with mean (SD) age of 20.6 (1.9) years. The age-adjusted prevalence (with 95% Confidence Interval) for ever use of tobacco, alcohol, and other substances was 34.2% (33.9, 34.5), 23.4% (23.2, 23.6), and 4.5% (4.4, 4.5), respectively. Loneliness and suicidal thoughts were reported by 237 and 35 youth men with age adjusted prevalence as 28.6%, 95% CI: 28.4–28.8 and 4.3%, 95% CI: 4.23–4.31, respectively. A total of 330 young men met serious injury in past one year (prevalence 39.3%, 95%CI: 39.01–39.67). Almost one-third of men (prevalence 30.6%, 95%CI: 30.34–30.85) had engaged in pre-marital sexual intercourse. Current substance use was found to be significant mediator for associations with socio-demographic variables studied for dependent variables viz. pre-marital sexual intercourse and serious injury.

**Conclusion:**

High prevalence of various risk behaviors and outcomes was found in young men aged 18–24 years in our rural setting. It is imperative that multi-component health intervention package be rolled out to address these.

## Introduction

Youth, the productive and dynamic section of the population, is considered the most valuable human resource for fostering economic, cultural and political development of a nation. The World Health Organization (WHO) defines youth as age group 15–24 years [1].This period is marked with immense physiological, psychological and behavioral changes coupled with varying patterns of social interactions and relationships. This also leads to engagement in multiple risk behaviors. [2,3]. According to the United Nations, about 16% of the world’s population is comprised of youth, and this proportion is higher in countries with increasing population like China and India [4]. As per Census 2011 in India, 19.1% of India’s population is in the age group of 15 to 24 years [5].

Most young people are presumed to be healthy; however, as per the World Health Organization, an estimated 1.2 million young people die each year. A much greater number of young people suffer from illnesses ‘behaviors’ which in later years can be related to their diseases [6,7]. In India, age-specific mortality rate in the age group of 15–24 years is 2.3/1000 population[4]. For age group of 15–39 years, 11.6% of total age-specific mortality rate is related to transport related injuries, and 15.4% is related to interpersonal violence [8].

According to the National Mental Health Survey in India, 13.5% participants in the age group of 18–29 years reported substance use, among which tobacco consumption is commonest. This proportion of substance use is higher in rural India [9]. Similarly, high risk sexual behavior among Indian youth is high. According to the National Family Health Survey-3 (2005–06), 7% of youth in India have two or more sexual partners, and only 36% of them have used condom during sexual intercourse [10]. Moreover, bundling of risk behaviors i.e., having multiple risk behaviors together is another important feature among youth [11]. Health needs of young men are accorded less attention than women, with focus on reproductive health, marriage and contraception. Tobacco, alcohol and drug use are more common in young men than women, with immediate and long term consequences along with linked effects on violence and injuries [12]. With paucity of data related to the extent of problematic behaviors in rural settings of India, we planned this study to investigate multiple risk behaviors (substance use, sexual behavior, use of helmets and seatbelts), outcomes (mental health, injuries and violence), and their related factors among young men (18–24 years) in rural north India. We report here the findings of this survey.

## Material and methods

### Ethics

The ethics clearance was taken through Institute Ethics Committee of All India Institute of Medical Sciences, New Delhi (IEC/91/2/2017). The study was done according to principles laid by Declaration of Helsinki. Written informed consent was obtained from the study participants. All the data forms were kept securely under lock and key with restricted access to the study investigators. Data entry was performed without having personal identification details and linkage was done through unique identification details for all study participants; the study database was password protected. All the information were kept confidential.

This was a community-based cross-sectional survey conducted in the Ballabgarh block of Faridabad district, Haryana, India. The sampling frame consisted of young men residing in 28 villages with total population of 99,965 [13]. The entire population was listed in our computer-based health management information system. Data was collected from November 2017 to January 2018. Young men in the age group 18–24 years residing in the study area who were willing to participate, and gave written consent were included in the study.

Sample size was calculated considering prevalence of current smoking in young men aged 15 years and above as 23% [14]. This was used as district level information, within which our study area was located, was available for this parameter only. Taking absolute precision of 3%, level of significance 5%, power of 80%, a minimum of 751 young men were required in the study. We also considered non-response rate of 14% (based on our experience of pilot study undertaken), 856 young men were considered for inclusion in the study.

A computer-based random list of 860 eligible young men was generated. House-to-house visit was made to contact all 860 selected individuals. During the first visit, contact numbers of all eligible individuals were collected. If a participant was not present, the likely time of his availability was ascertained, and second house visit was made accordingly. A maximum of two house visits were made to contact the randomly selected participant before declaring him non-contactable.

A pre-tested semi structured interview schedule was administered to collect information regarding socio-demographic details (age, years of schooling, marital status, current occupation, education of parents, occupation of parents, total monthly family income), substance use behavior (current use of tobacco- smoke and smokeless forms, pattern of smoke and smokeless tobacco use among past users, pattern of present and past use of alcohol and use of substances other than tobacco and alcohol); injury (frequency, type of injuries, use of helmet during bike riding, use of seat belts during car driving, drinking and driving) & violence (possession of weapon, physical fight and bullying); mental health (loneliness, suicidal thoughts and suicidal attempts) and sexual behaviors (age at first sexual intercourse, number of sexual partners, sexual intercourse under influence of substance, safe sex practice, use of contraceptives to prevent unwanted pregnancies, unwanted sexual contact and intercourse). The instrument was prepared through a review of validated instruments already in use globally for understanding youth risk behaviors [15,16]. Since these were validated and reliable questionnaires and with previous use in Indian setting, a separate attempt to validate these was not done as part of this study. Data were collected by trained physicians who were well versed with the content of the interview schedule. The study interview schedule is produced within S1 File.

Pilot testing of the study procedures was done among young men aged 15–24 years in a separate rural setting adjacent to study area. The main focus of the pilot study was to standardize the procedure for conducting interviews, especially feasibility of asking sensitive questions in field settings, and also to finalize the interview schedule. During pilot testing, it was observed that some young men were not comfortable in answering sensitive questions in their family setting. Based on this finding of pilot testing, it was decided to interview study participants in nearby health centers if there were no secluded room in the house to maintain confidentiality. It was also found that obtaining consent from parents was difficult for the study participants aged 15–17 years as the study contained components regarding substance use and sexual behavior. Realizing this, the final study was planned for the age groups 18–24 years, instead of 15–24 years. During interview, measures were taken to maintain privacy so that reliable responses could be elicited from the participants. To ensure quality, ten percent of all participants were selected randomly and re-visited, and their responses were verified.

**Fig 1:**
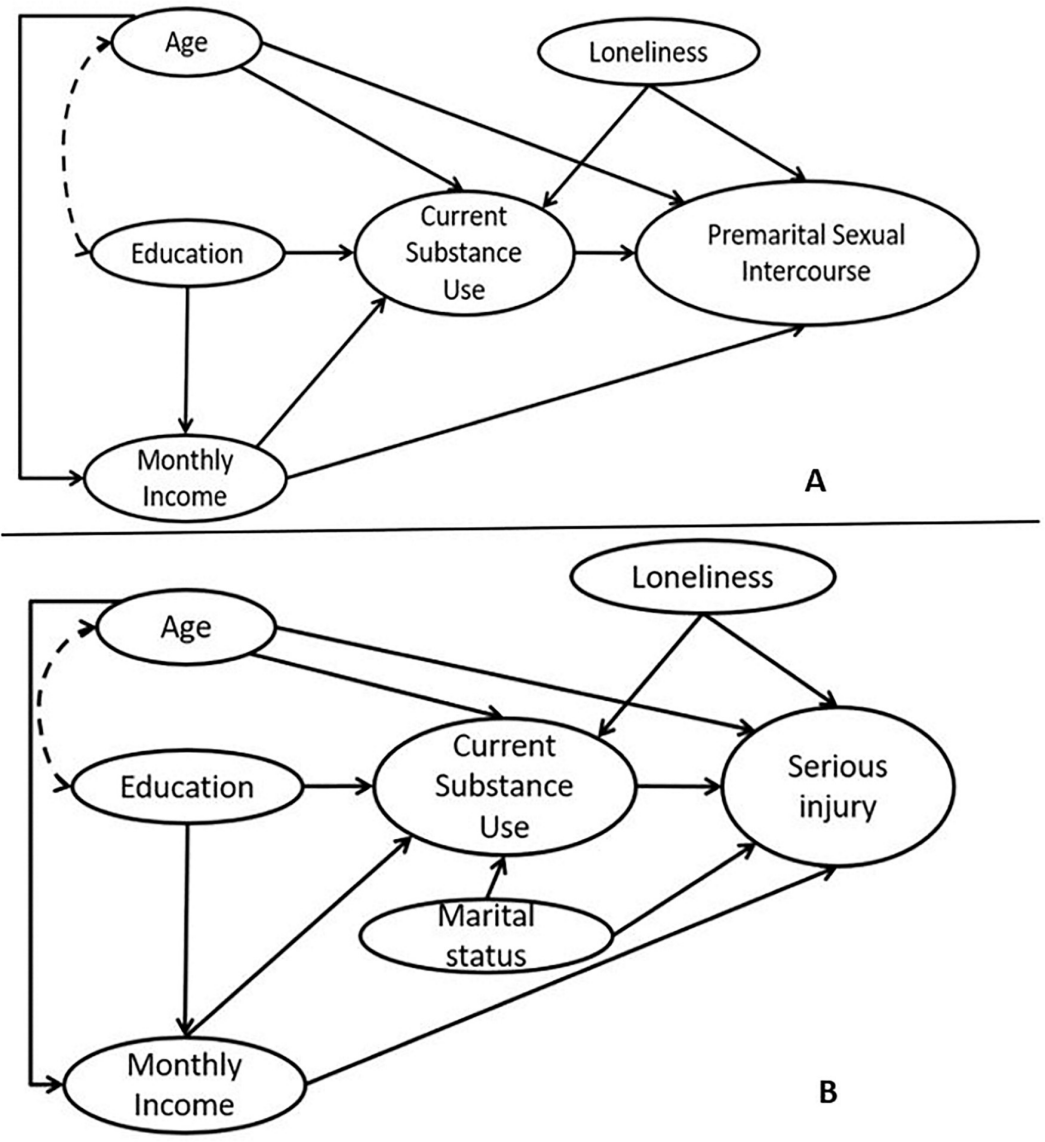
Conceptualized model with hypothesized paths for A. premarital sexual intercourse B. serious injury. The model depicts possible paths of independent variables for their relationship with dependent variable i.e. premarital sexual intercourse and serious injury respectively.

Data were entered in Microsoft Excel 2013 and analyzed using STATA software version 12.0 (StataCorp LP, College Station, Texas, USA). Categorical variables and continuous variables were expressed as number (percentage) and mean ± SD respectively. Age-adjusted prevalence (95% CI) estimates for the health behaviors and outcomes were reported. The independent variables considered were age, education, marital status, monthly family income, loneliness and current substance use and the dependent variables were premarital sexual intercourse and serious injury. Mediation analysis was carried to find the relationship of age, marital status, education, monthly family income, loneliness and current substance use with premarital sexual intercourse and serious injury (ordinal variables). The correlation between independent and dependent variables were calculated using Pearson correlation coefficient. The hypothesized paths in the conceptual framework (Fig 1) were tested using series of regression analysis and standardized path coefficients were calculated. The direct effects were standardized path coefficients itself (p_d,i_ denote path (p) coefficient for path between dependent variable (d) and independent variable (i)) and it represent the magnitude of influence of one variable over the other in the path model. The indirect effects were the sum of all compound paths calculated using path coefficients of simple paths in the model. P-value of <0.05 was considered as statistically significant.

### Operational definitions

1. Young men: Men in age group 18–24 years.
2. Illiterate: An individual who was unable to read and write any single language.
3. Substance: Tobacco, alcohol and other drugs [17].
4. Other drugs: Harmful or hazardous use of psychoactive substances and illicit drugs other than tobacco and alcohol, like ganja, charas, brown sugar, cocaine, bhang, etc.
5. Smokeless tobacco: Tobacco that is chewed or snuffed rather than smoked by user.
6. Ever users of substance: An individual who used tobacco, alcohol or other drugs at least once in his life time.
7. Current users of substance: An individual who was using tobacco, or alcohol or other drugs for past one month.
8. Heavy Drink: Individuals with more than one occasion of having six or more standard drinks (30 ml of drink) in a single drinking session in last one month.
9. Serious injury: Any injury that leads to missing of at least one full day of usual activity such as school, sports or a job [15].
10. Weapon: Gun or Knife [15].
11. Loneliness: A self-perception of being alone and isolated.
12. Suicidal ideation: An idea to end one’s own life.

## Results

Of the randomly selected 860 young men, a total of 836 participated in the study. Twenty one young men were non-contactable, and three declined to give consent. The response rate was 97%. The mean (SD) age of young men was 20.6 (1.9) years, and mean (SD) years of schooling was 11.8 (2.8) years. Majority of the men were unmarried (86.4%). The socio-demographic characteristics of the participating young men are shown in Table 1.

**Table 1:** Socio-demographic details of study participants.

Characteristics		Number (%) (n = 836)
Age (in years)	≤20	429((51.3)
	>20	407(48.7)
Marital status	Unmarried	723(86.5)
	Married	113(13.5)
Education status (years of schooling)	≤10 years	269(32.2)
	>10 years	567(67.8)
Occupation category of participants	Student	430 (51.4)
	Unemployed	84 (10.1)
	Employed	322 (38.5)
Mother’s education	Illiterate	404 (48.3)
	Literate	432 (51.7)
	Mean (SD) years of schooling for literate (n = 432)	7.2 (2.5)
Father’s education	Illiterate	122 (14.6)
	Literate	714 (85.4)
	Mean (SD) years of schooling for literate (n = 714)	9.2 (2.9)
Mother’s occupation	Housewife	745 (89.1)
	Involved in farming	17 (2.0)
	Day laborer	16 (1.9)
	Others	58 (6.8)
Father’s occupation	Farmer	263 (31.5)
	Day laborer	98 (11.7)
	Self employed	85 (10.2)
	Private job	78 (9.3)
	Others	312 (37.3)
Median(IQR) monthly family income		Rs.12,000 (8750, 20000)

SD: Standard Deviation, IQR: Inter Quartile Range

### Substance use

The age-adjusted prevalence (with 95% Confidence Interval) for ever use of tobacco, alcohol and other substances was 34.24% (33.96, 34.52), 23.38% (23.19, 23.58), and 4.5% (4.47, 4.55), respectively. The age-adjusted prevalence of current use of any form of substance was 32.3% (95% CI: 31.6, 33.0).The prevalence of current tobacco use and current smoking was (29.69%, 95% CI: 29.44, 29.93) and (27.59%, 95% CI: 27.36, 27.82), respectively. Mean (SD) age of initiation of smoking among current users were 17.6 (3.0) years. Among the 232 current smokers, 29.69% (95% CI: 29.44, 29.93) tried to stop smoking. Thirty-one current smokers (13.4%) were advised to stop smoking during their last visit to doctor or health worker. Currently, 8.29% (95% CI: 8.22, 8.36) of young men were using smokeless tobacco; whereas 6.20% (95%CI: 6.14–6.25) of young men were using both types of tobacco products currently. Details of substance use are provided in Table 2.

**Table 2:** Pattern of substance use among young men.

Characteristics	Number	Age Adjusted Prevalence (95% CI)
**Tobacco**		
Ever tobacco user (n = 836)	285	34.2 (33.9–34.52)
Any form of current tobacco user (n = 836)	248	29.7 (29.4–29.9)
Current smoker (n = 836)	232	27.6 (27.4–27.8)
Mean (SD) age of start of smoking (years) (n = 232)	17.6 (3.0)	-
Past smoker (n = 604)	39	6.9 (6.9–7.0)
Current user of smokeless tobacco product (n = 836)	64	8.3 (8.2–8.4)
Past user of smokeless tobacco product (n = 772)	06	0.84 (0.83–0.84)
Current user of both types of tobacco	52	6.2 (6.1–6.3)
**Alcohol**		
Ever alcohol user (n = 836)	194	23.4 (23.2–23.6)
Alcohol use in past 12 months (n = 836)	168	20.1 (19.9–20.2)
Alcohol use in last one month (n = 836)	88	10.4 (10.3–10.5)
Heavy Drinking (n = 88)	29	35.9 (35.6–36.2)
**Substance Use other than tobacco and alcohol**		
Ever Use (n = 836)	27	4.5 (4.4–4.5)
Current Use (n = 836)	25	3.0 (2.7–3.3)
**Any form of Substance Use**		
Ever Use of any form of substance (n = 836)	375	44.9 (44.5–45.2)
Current use of any form of substance (n = 836)	266	32.3 (31.6–33.0)

### Mental health

Loneliness and suicidal thoughts in past 12 months were reported by 237 and 35 young men with age-adjusted prevalence as 28.60% (95% CI: 28.35, 28.83) and 4.27% (95% CI: 4.23, 4.31), respectively. Seven (0.80%, 95% CI: 0.79–0.81) young men attempted suicide during the same duration.

### Injuries and violence

The age-adjusted prevalence of serious injury in past one year was 39.34% (95%CI: 39.32–39.37). Among the 330 young men who reported serious injury, road traffic accident was the most common cause (36.08%) of serious injury, followed by work related injury (29.46%). Details are presented in Table 3. About one-fifth (18.68%), and one-tenth (8.72%) young men had never used helmet and seat belt, respectively during riding a bike or car in last one year. Almost 17% young men rode a car or other vehicle in last one month when the driver was drinking alcohol at least on one occasion (n = 759). Moreover, around 6% young men drove a car or other vehicle after drinking alcohol at least on one occasion (n = 741).

**Table 3:** Pattern of injuries, violence and related behaviors among young men.

Characteristics		Number	Age-Adjusted Prevalence (95% CI)
Participants with at least one episode of serious injury in last one year(n = 836)		330	39.3 (39.3–39.4)
Causes of serious injury (Multiple responses) (n = 330)	Work related injury	96	29.5 (29.2–29.7)
	Sports related injury	85	24.7 (24.5–24.9)
	Road Traffic Accident	117	36.1 (35.8–36.4)
	Physical fight	62	19.8 (19.6–19.9)
	Others	23	13.9 (13.9–14.1)
Use of helmet (n = 836)	Never user	158	18.7 (18.5–18.8)
	Ever user	648	77.8 (77.2–78.5)
	Not applicable	30	3.5 (3.4–3.5)
Use of seat belt (n = 836)	Never user	71	8.7 (8.6–8.8)
	Ever user	384	46.5 (46.1–46.8)
	Not applicable	381	44.8 (44.5–45.2)
Carried weapon		13	0.84 (0.83–0.85)
Engaged in physical fight		145	19.8 (19.6–19.9)

Possession of weapon in past one month was reported by 13 youth with age-adjusted prevalence of 0.84% (95% CI: 0.83, 0.85). Twenty-seven (3.23%, 95%CI: 3.20, 3.25) youth reported of being threatened or injured by someone in past one month. In last one year, 145 (19.75%, 95% CI: 19.58, 19.91) youth reported to be engaged in physical fight. More than half of the youth (56.7%) reported to have experienced someone intentionally rude or insulting (verbal abuse) to them in last one year.

### Sexual behavior

The age-adjusted prevalence of sexual intercourse during lifetime was 38.17%, 95% CI: 37.86–38.49. Two hundred and six (30.59%, 95% CI: 30.34–30.85) young men had engaged in pre-marital sexual intercourse. The mean (SD) age at first sexual intercourse was 18.3 (2.5) years. Seventeen (5.5%) and one hundred and eight (35.1%) young men had their first sexual intercourse before the age of 15 years and 18 years, respectively. Other details are mentioned in Table 4. Among the sexually active young men (n = 308), 42.94% (95% CI: 42.58, 43.29) had more than one sexual partner during their life time. Twenty-nine (6.93%, 95% CI: 6.87, 6.99) young men reported to have consumed alcohol or used a drug before sexual intercourse (n = 308).Slightly more than one-fourth (27.47%, 95% CI: 27.24, 27.69) of young men used condom during last sexual intercourse (n = 308). However, almost half of the young men (53.58%, 95% CI: 53.13, 54.02) did not use any contraceptive method during last sexual intercourse. When the young men were enquired about ever experienced sexual abuse, 6.93% (95% CI: 6.87, 6.99) of them reported that someone had touched their private parts without consent, and five (0.57%, 95% CI: 0.57, 0.58) of them reported to have sexual intercourse without their consent.

**Table 4:** Pattern of sexual behaviors of young men.

Characteristics		Number	Age Adjusted Prevalence (95% CI)
Had sexual intercourse		308	38.2 (37.9–38.5)
Pre-marital sexual intercourse (n = 722)		206	30.6 (30.3–30.9)
Mean (SD) age at 1^st^ sexual intercourse (years) (n = 308)		18.3 (2.5)	-
Median (IQR) number of sexual partners (n = 308)		1 (1, 2)	-
Median (IQR) number of sexual partners in past three months (n = 308)		1 (0, 1)	-
Consumed alcohol/under influence of any drug during sexual intercourse (n = 308)		29	6.9 (6.8–6.9)
Contraceptive method used to prevent pregnancy (n = 308)			
Contraceptive method used to prevent pregnancy (n = 308)	Barrier Method (Condom)	87	27.5 (27.2–27.7)
	Other methods	61	18.9 (18.8–19.1)
	None	160	53.5 (53.1–54.0)
Any one touched private parts without consent (n = 836)		58	6.9 (6.8–6.9)
Sexual intercourse without consent (n = 836)		05	0.57 (0.57–0.58)

### Relationships with selected health behaviors and outcomes

We examined the relationship of socio demographic factors and substance use on premarital sexual intercourse and serious injury. Education, marital status and monthly family income were significantly and positively correlated with age; current substance use was significantly and positively correlated with age, marital status and loneliness and negatively correlated with education and monthly family income whereas loneliness was not correlated with age, education, marital status and monthly family income (shown in Table 5).

**Table 5:** Correlation between study variables and premarital sexual intercourse and serious injury.

Correlation	1	2	3	4	5	6	7	8
1. Premarital sexual intercourse	1.00							
2. Serious injury	-	1.00						
3. Age	0.09*	-0.01	1.00					
4. Education	0.02	-0.05	0.25*	1.00				
5. Marital status	-	-0.06	0.32*	0.01	1.00			
6. Monthly family income	-0.02	-0.03	0.09*	0.13*	0.12*	1.00		
7. Loneliness	0.18*	0.12*	0.01	-0.01	-0.04	-0.05	1.00	
8. Current substance use	0.20*	0.15*	0.14*	-0.19*	0.13*	-0.07^$^	0.19*	1.00
Mean	0.60	0.48	20.6	11.7	0.14	18450	0.28	0.32
SD	1.6	0.68	1.9	2.8	0.34	22326	0.45	0.47

*p<0.01 and

^$^ p<0.05

### Mediation analysis

**Fig 2:**
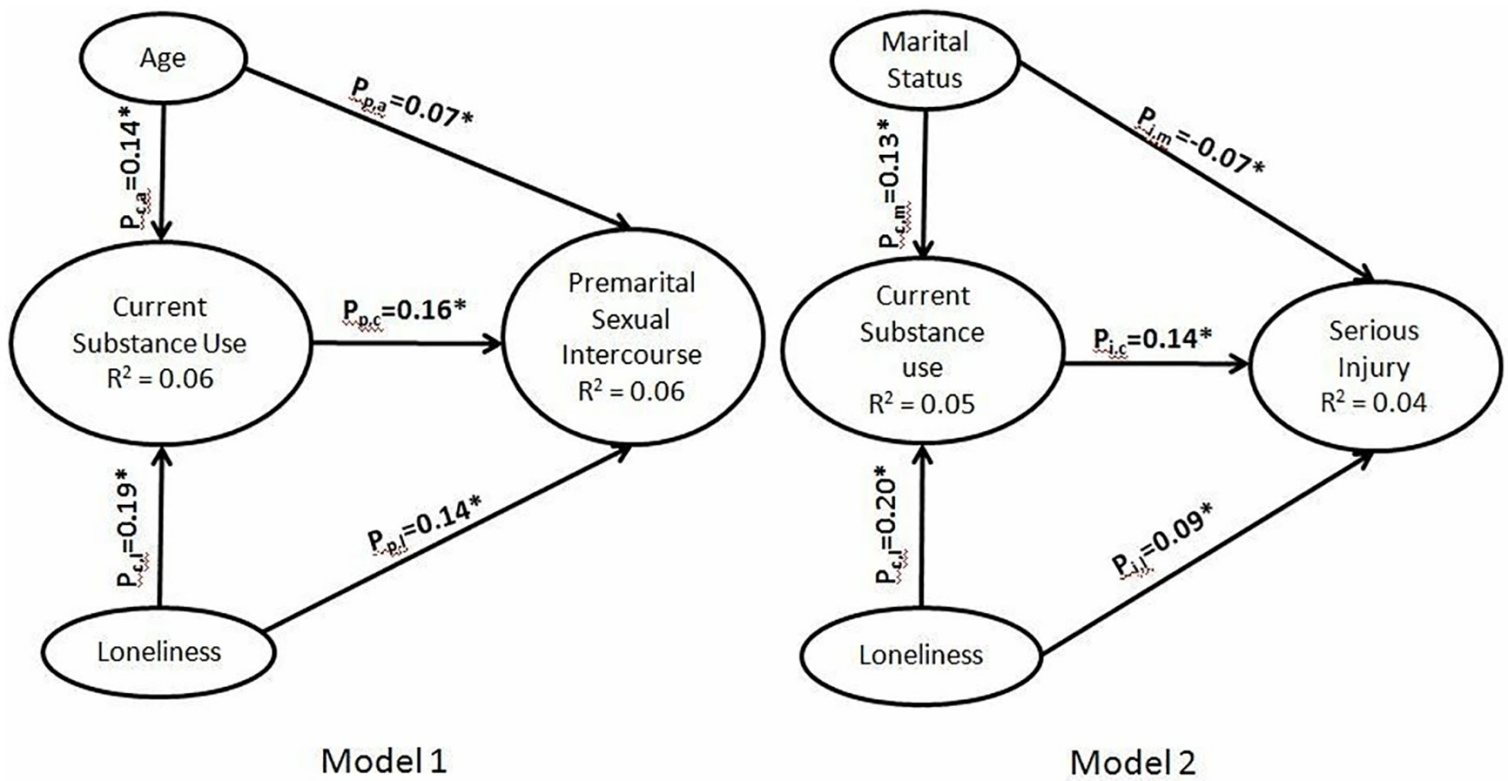
Standardized path estimates. Model 1 depicts standardized path coefficients between significant independent variables and premarital sexual intercourse. Model 2 depicts standardized path coefficients between significant independent variables and serious injury. * p<0.001. R^2^– proportion of variance explained.

We tested the conceptualized framework with hypothesized paths (shown in Fig 1) using two path analysis, viz., i) to study the relationship between socio-demographic factors and substance use on premarital sexual intercourse and ii) on serious injury. Series of regression analysis and some hand calculations led to standardized path coefficients (shown in Fig 2), direct, indirect effects, non-causal and total effects. For each of model 1 and model 2 we needed two regression analysis—one with premarital sexual intercourse regressed onto age, current substance use and loneliness and other with current substance use regressed on to age and loneliness for model 1; whereas for model 2, one with serious injury regressed onto marital status, current substance use and loneliness and other with current substance use regressed on to marital status and loneliness. In both the model i) and ii) we found two endogenous variables—diagrammed as being influenced by other variables, model i) premarital sexual intercourse and current substance use and in the model ii) Serious injury and current substance use.

As shown in Fig 2. model 1, age (p_p,a_ = 0.07, p = 0.001), current substance use (p_p,c_ = 0.16, p = 0.001) and loneliness (p_p,l_ = 0.14, p = 0.001) had a significant direct effect on premarital sexual intercourse; age (p_c,a_ = 0.14, p = 0.001), and loneliness (p_c,l_ = 0.19, p = 0.001), had a significant direct effect on current substance use. The other variables education and monthly family income diagrammed in Fig 1 were not statistically significant in the initial regression analysis removed from the subsequent model 1 of Fig 2. Age and loneliness also had a very minimal non-significant indirect effect on premarital sexual intercourse through current substance use (age, current substance use, premarital sexual intercourse; p_c,a_*p_p,c_ = 0.022 and loneliness, current substance use, premarital sexual intercourse; p_c,l_*p_p,c_ = 0.030) and total effects of 0.09 (p<0.001) and 0.17 (p<0.001). Thus our hypothesis that current substance use acted as a mediator variable for premarital sexual intercourse, as indicated in the conceptual framework earlier, was true.

For serious injury, shown in Fig 2. model 2, marital status (p_i,m_ = -0.07, p<0.001) had a significant negative direct effect indicating married youth having less serious injury than unmarried youth. Current substance use (p_i,c_ = 0.14, p = 0.001) and loneliness (p_i,l_ = 0.09, p = 0.001) had significant direct relationship with serious injury as seen with premarital sexual intercourse. The influence of marital status and loneliness through current substance use on serious injury was 0.018 (p_c,m_*p_i,c_) and 0.028 (p_c,l_*p_i,c_) respectively, were not significant and the total effects of -0.052 (p = 0.001) and 0.118 (p = 0.001) were statistically significant. Here also, our hypothesis that current substance use acted as a mediator variable for serious injury, as indicated in the conceptual framework earlier, was found to be true.

## Discussion

This was a community-based cross-sectional assessment of multiple health behaviors of young men aged 18–24 years in a rural setting of north India. This age group was considered for this study instead of complete age group of youth (15-24yrs) owing to sensitive nature of information enquired in community settings, based on experience of our pilot study as stated earlier. The prevalence of these behaviors have been found to be substantially high. The current tobacco use among the youth was 29.7%. Our finding is consistent with the sub-nationally representative youth survey undertaken in six states of India, that reported current tobacco consumption in youth aged 15–24 years as 31% [18], and lower than overall prevalence of current tobacco use in men for the state of Haryana (39%) as per the recent round of Global Adult Tobacco Survey undertaken in adults aged 15 years and above [19]. The same survey reported 32% prevalence for rural India. The variations are noted owing to differences in study setting, age group, methodology, and background profile of youth that is markedly different in parts of the country. Lifetime alcohol use among youth was 24%. It has been reported that, in Haryana state, 24.2% rural population in the age group of 15–49 years consumed alcohol [20]. A study among north Indian college students of similar age group reported higher prevalence (53%) of alcohol consumption[21]. Rural residence and living with family were the possible reasons behind lower prevalence of alcohol consumption in the current study. About 4.5% young men gave history of using other substances. The findings of the current study are consistent with another Indian study conducted among north Indian male college students of similar age group [22]. Use of alcohol, tobacco or substances other than tobacco and alcohol among young men were found significantly associated with socio-demographic variables. This finding was consistent with available literature [23]. In our study, current substance use influenced and mediated the effect of other socio-demographic variables on key outcomes viz premarital sexual intercourse and serious injury and emerged out to be an important risky behavior for youth health.

Road Traffic Accident RTA (35.5%) was the most common cause of serious injuries in our study. An Indian study has reported that RTA, physical fight and falls were common causes of injury among youth in their study [24]. The current study reported low usage of helmets (18%) and seat belts (14%) coupled with substantial young men reporting driving vehicles after consuming alcohol (17%). It has been reported that, drunken driving, failure to use helmets and seat belts are major reasons for RTA in India [25]. Involvement of young men in various sports-related exercises was the possible reason behind high sports-related injuries (26%) in the present study [26]. Most of the young men belonged to agrarian background and probably engaged in farming activities, which probably was the reason behind high work-related serious injuries (29%) in this study [27]. Physical fight was one of the major reasons for serious injury in our study (18%). An Indian study reported similar prevalence of serious injuries among youth due to physical fight [28]. Youth violence is reported in multiple ways. A high proportion of young men (57%) reported verbal abuse. Almost 0.8% young men carried weapon in last one month. This prevalence was lower than the prevalence reported in another Indian study, where almost 12% of the participants were found to carry weapon in similar time frame [28]. Unlike the current study, the latter study was done among urban school/college going adolescents (14–19 years). A possible explanation is that with increasing age, young people become more mature and gain skills to solve conflicts in non-violent ways. Moreover, in rural areas people live in a strong social fabric which could have played a preventive role. Current substance use was found to be significantly associated with injuries and was found to be mediating the effect of other variables studied viz marital status and loneliness in our analysis. Substance use had been linked to trauma in young people and consistently had been found to be resulting in more frequent injuries and serious injuries [29,30].

Perception of loneliness is an important surrogate of mental health wellbeing among youth. Loneliness may predispose to multiple mental health disorders in the long term, including suicidal ideation and substance abuse [31]. In the current study, 28.6% young men reported feeling loneliness in past one year. In South East Asian (SEA) countries, the reported prevalence of loneliness among youth was found to range from 6.7% in Indonesia to 15.5% in Maldives [32]. A global school-based student health survey in India had reported that almost 7.4% youth experienced loneliness in past one year [33]. Both studies were done among adolescents less than 18 years. Though mental health problems appear in the adolescence, they becomes more prevalent in young age. Moreover, in this age group uncertainty of future, academics, financial issues and relationship problems are more prevalent which could be the possible explanation behind high burden of loneliness found in our study. In the current study, current substance use was found to be positively associated with loneliness. This is consistent with other studies where loneliness, and alcohol and cannabis use have been found to be positively associated as their use served as coping mechanism [34]. The National Mental Health Survey-2016 (NMHS-2016) had reported that around 5.5% Indian rural population had suicidal risk which included suicidal ideation and thoughts of attempting suicide. Finding of the current study is almost similar to the findings of NMHS-2016 [35]. Though NMHS was done among age group more than 18 years, a possible explanation for similar prevalence is that, with increasing age people become more mature and handle life pressure more efficiently. In the current study, 0.8% of young men attempted suicide, i.e., one fifth of young men who had suicidal ideation. Another study from urban north Indian setting reported that 13.8% men had suicidal ideation, and 4.1% men attempted suicide [36]. Strong social bonding among rural population might be one of the reasons for lower prevalence of suicidal attempts in the current study. The reported prevalence of suicidal ideation among adolescents from other countries in SEA region concurs with the findings of our study. However, the prevalence of suicidal attempt was found to be much higher than current study [32]. Other differences in our study could be ascribed to the difference in age profile of youth and community-based random sampling.

The prevalence of pre-marital sexual intercourse 30.6% was higher than the studies conducted in other parts of India in the similar age group [23,37,38]. Another worrisome finding was that only 27.5% youth had used condom during last sexual intercourse. Our finding concurs with another study that also reported lower prevalence of condom use among youth [23,37]. The same national level survey among youth of various states in India had reported that 25% of youth who experience pre-marital sexual intercourse had more than one partner [23]. About 6.9% of young men consumed alcohol or were under the influence of drug before their last sexual intercourse. It has already been reported that taking alcohol or other substances increases high risk sexual behavior and vulnerability to sexually transmitted diseases [39]. Mean age of young men at first sexual intercourse was 18.3 years. This finding is similar to a study among youth in Karnataka [40]. However, mean age of first sexual intercourse in our study was lower than the findings of a national level survey [41]. More than half of the young men did not use any contraceptive method during last sexual intercourse. As per National Family Health Survey 4 report, around 62% rural population were currently using any contraceptive method [20]. The NFHS-4 reported use of family planning method only among married population aged 15–49 years. The unmarried population who usually do not engage into regular sexual activity, and young married population who possibly do not yet plan for family planning, accounted for lower prevalence of contraceptive use in the current study. Almost 6% young men reported that someone touched their private parts without their consent, and 0.57% young men had experienced sexual intercourse against their will. A higher prevalence of sexual abuse and sexual intercourse had been reported in a study conducted in the Indian state of Goa [42]. The latter study was done among school-going adolescents (both male and female). Higher vulnerability of female population to sexual abuse, and higher curiosity of sexuality in younger age were the possible reasons behind high prevalence in the latter study. Conservative attitude towards sexuality in the current study area could have played an important role in the lower prevalence of sexual abuse in our study. Possibility of under reporting in the current study could not be ruled out. In India, sexual violence is common against women. However, sexual violence against men goes unnoticed and unreported most of the time. Experiencing sexual violence may give rise to mental illnesses, and even makes the victim vulnerable to suicidal ideation and committing suicide. The chance of experiencing sexual activity rises with increase in age [43]. This was the possible reason behind association between pre-marital sexual intercourse and higher age group. Studies across globe have found that tobacco use, alcohol use, multiple substance use and loneliness were associated with pre-marital sexual activity [43,44, 45]. Alcohol consumption has been linked to risky sexual behavior in young people through multiple explanations influencing perceptions, loss of inhibitions and memory [46]. In our study current substance use was found to have mediating effect of other socio-demographic variables on pre-marital sexual intercourse and this effect was found significance and is an important finding of our study.

A substantially high proportion of young men were indulging in problem and risky behaviors [substance use (39%)], and outcomes [pre-marital sexual intercourse (31%) and serious injury (39%)] which are amenable to preventive and treatment interventions. It is imperative that the young men in our study area are sensitized to evidence-based interventions that result in their overall positive health development encompassing structural, community and individual components targeting multiple behaviors. There is a need to raise awareness among youth about harmful effects associated with risky behaviors and ways to prevent these. As current substance use in any form significantly mediated the effect of other associated factors for both the outcomes studied- premarital sexual intercourse and serious injury. It is imperative that interventions targeting young men should aim at modifying current substance use. This behavior seems to have high potential in affecting other risky outcomes as studied here. One-third of our young men attempted tobacco cessation, an important finding for action and these young men should be adequately supported by the health system for tobacco cessation and prevention of relapses. Counselling and support services are warranted to tackle alcohol and substance use among youth. It is equally critical that young men be adequately informed about harms imposed through unsafe sex; condom supplies to be ensured for sexually active young men for increasing the contraceptive use during sexual contact, which was found to be low, as 54% young men did not report using contraceptive method during last intercourse. A wider approach for screening and brief intervention provision through youth-friendly services is required to address health issues of young men in our area. There is linkage among different factors and thus comprehensive approach to tackle multiple issues is warranted. Keeping in mind, high proportion of serious injury (39%), verbal abuse (57%) and physical fights (20%), youth violence prevention programmes also merit planning and programmatic response.

This was one of the first studies in Ballabgarh Block in north India to best of our knowledge which assessed health risk behaviors and outcomes among young men. It utilized a methodology that could study multi-faceted health behaviors and thus was comprehensive in its approach. The sampling methodology was community-based and employed random selection of youth from study area, enhancing representativeness of study findings. The study had some limitations. Our sample size was computed using tobacco use only, though we studied multiple behaviors among young men with some of behaviors quite prevalent low and would require higher sample size. All information was gathered as self-reports. Social desirability bias cannot be ruled out owing to sensitivity of issues enquired, though sufficient attention was accorded to rapport building, privacy and assurance for confidentiality by study team members while gathering data in a community setting. The bias could under-estimate (substance use) and over-estimate (sexual behavior) the prevalence estimates. We performed mediation analysis using cross-sectional data, which is limited in its scope unlike a longitudinal study that gives advantage of establishing direction of relationship and causality. Also, the dependent variables analyzed viz. premarital sexual intercourse and serious injury were ordinal in nature. The study was undertaken in rural settings, thus limiting the scope of generalizability to similar settings and included young men only. We considered men to be neglected in youth health agenda, and thus focused the study targeting this segment of youth. Future studies related to these issues can dwell on several of these in greater depth like in our study, we used only loneliness as a surrogate measure for adverse mental health status. Detailed enquiry for common mental health disorders like anxiety and depressive disorders can be examined using standardized psychiatric assessment scales. Also, National youth policy in India, considers youth within age group 15–29 years [47], this can be considered as age group for studying youth health behaviors based on feasibility and setting to inform policy and programme practice.

## Conclusion

High prevalence of problem behaviors and outcomes were seen in young men aged 18–24 years in a rural area of north India. Higher educational status and family incomes were inversely associated with risk behaviors. Positive associations were found with higher age, marriage, current substance use and loneliness. Comprehensive package of services targeting multiple risk behaviors and outcomes is imperative for addressing young men health needs in our setting. The prevalence estimates generated for multiple health behaviors and outcomes in the current study can serve as baseline indicators for rolling out youth health interventions in this setting and further monitoring the progress in future.

## Supporting information

S1 File Survey interview schedule. The file contains questions enquired from the participants as part of the study in various domains contextual to the study.
(PDF)

S2 File STROBE checklist. The file contains items included as per STROBE checklist and the page numbers where relevant information is included.
(DOC)
