# Correction: Health behaviors, outcomes and their relationships among young men aged 18-24 years in a rural area of north India: A cross-sectional study

**DOI:** 10.1371/journal.pone.0221493

**Published:** 2019-08-15

**Authors:** Sumit Malhotra, Shashi Kant, Farhad Ahamed, Ramashankar Rath, Mani Kaladivani, Sanjeev Kumar Gupta, S. Ramadass, Vineet Kumar Pathak, Abhishek Jaiswal, Raghavan Parthasarathy, Bhabani Prasad Acharya, Vignesh Dwarakanathan

The tenth author’s name is incorrect. The correct name is Raghavan Parthasarathy.
