# Supplementary material for: Health behaviors, outcomes and their relationships among young men aged 18-24 years in a rural area of north India: A cross-sectional study
Source: PLoS One. 2019 Jul 26;14(7):e0220285. doi: 10.1371/journal.pone.0220285 (PMC6662032; doi:10.1371/journal.pone.0220285)
Supplement: S1 File — The file contains questions enquired from the participants as part of the study in various domains contextual to the study. (PDF) [file pone.0220285.s001.pdf]

Health behaviors and outcomes among youth men aged 18-24 years in a rural area of north India

Survey Interview Schedule

Interview to be conducted in **private** to ensure confidentiality of the information provided.

**Individual ID**		**Date of Interview:**
	**Section A (General Information)**<br>**(सामान्य जानकारी)**	
**1**	Age / उम्र	
**2**	Education: Years of Schooling/ कितने साल की स्कूली शिक्षा	
**3**	Marital status 1.Unmarried 2.Married	
**4**	Current Occupation / वर्तमान व्यवसाय	
**5**	Mothers Education / माँ की शिक्षा	
**6**	Fathers Education / पिता की शिक्षा	
**7**	Mothers Occupation / मां का व्यवसाय	
**8**	Fathers Occupation / पिता का व्यवसाय	
**9**	Total Monthly Family Income/ पारिवारिक आय (या) कमाई (monthly)	
**10**	Type of Mobile possessed & using: (1: Smart phone, 2: Non smart phone, 3: Not using mobile)	
	**Section B (Cigarette, Tobacco Use, Alcohol and Drugs)**<br>**(सिगरेट, तम्बाकू उपयोग, शराब और ड्रग्स)**	
**1**	Do you currently smoke any tobacco products, such as cigarettes, Beedi, Hukka, Cigar, Pipe?<br>क्या आप अभी किसी भी तम्बाकू उत्पादों का उपयोग करते हैं जैसे सिगरेट, बीडी, हुक्का, सिगार या पाइप?	☐ Yes<br>☐ No<br>**if No go to Q.4**
**2**	During the past 12 months, have you tried to stop smoking?<br>पिछले 12 महीनों में, क्या आपने कभी धूम्रपान बंद करने की कोशिश की थी?	☐ Yes<br>☐ No
**3**	During any visit to a doctor or other health worker in the past 12 months, were you advised to quit smoking tobacco?<br>पिछले 12 महीनों में, क्या आपको किसी डॉक्टर या स्वास्थ्य कर्मचारी ने धूम्रपान बंद करने की सलाह दी?	☐ Yes<br>☐ No
	**If no history of smoking currently, following questions to be asked:**	
**4**	In the past, did you ever smoke any tobacco products?<br>क्या आपने भूतकाल में (पहले कभी) किसी भी तम्बाकू उत्पादों का प्रयोग किया था जैसे सिगरेट, बीडी, हुक्का, सिगार या पाइप (हाँ/ना)	☐ Yes<br>☐ No
	**Smokeless Tobacco**	
**5**	Do you currently use any smokeless tobacco products such as *[snuff, chewing tobacco, betel]*?<br>क्या आप अभी कोई धूम्ररहित (बिना धुआं वाले) तम्बाकू उत्पादों का प्रयोग कर रहे हैं जैसे सुंघनी, चबाने वाला तम्बाकू, पान, इत्यादि ?	☐ Yes<br>☐ No
	**If no history of using smokeless tobacco currently, following questions to be asked:**	
**6**	In the past, did you ever use smokeless tobacco products such as *[snuff, chewing tobacco]*?<br>क्या आपने भूतकाल में (पहले कभी) कोई धूम्ररहित (बिना धुआं वाले) तम्बाकू उत्पादों का प्रयोग किया हैं (जैसे सुंघनी, चबाने वाला तम्बाकू,आदि)	☐ Yes<br>☐ No
**7**	Have you ever consumed any alcohol such as beer, wine, spirits?<br>क्या आपने कभी भी शराब का सेवन किया हैं (जैसे बीयर, वाइन , देसी शराब, इत्यादि)	☐ Yes<br>☐ No<br>**if No go to Q.12**
**8**	Have you consumed any alcohol within the past 12 months?<br>पिछले 12 महीनों में क्या आपने शराब का सेवन किया हैं ?	☐ Yes<br>☐ No
**9**	Have you consumed any alcohol within the past 30 days?<br>पिछले 30 दिनों में क्या आपने शराब का सेवन किया हैं?	☐ Yes<br>☐ No
**10**	During the past 30 days, on how many occasions did you have at least <u>one standard alcoholic drink</u>?<br>पिछले 30 दिनों में, आपने कितने दिन में कम से कम एक बार (30 ml) पिया होगा?	— — —
**11**	During the past 30 days, how many times did you have six or more <u>standard drinks</u> in a single drinking occasion?<br>पिछले 30 दिनों में आपने एक बार में 6 या 6 से ज्यादा पेग (30 ml) कितने बार लिए थे?	— — —
**12**	Have you ever used drugs such as ganja, charas, brown sugar, or cocaine? क्या आपने कभी गांजा, चरस, ब्राउन शुगर या कोकेन जैसी नशीले पदार्थो का सेवन किया है?	☐ Yes<br>☐ No<br>**If no go to section D**
**13**	How often in the last 30 days have you taken drugs?<br>आपने पिछले 30 दिनों में कितनी बार ड्रग्स ली? बिल्कुल नहीं / पिछले 4 हफ्ते में एक बार या दो बार / सप्ताह में एक बार / सप्ताह में एक बार से ज्यादा बार/ हर रोज़	☐ Not at all<br>☐ Once or twice in last 4 weeks<br>☐ Once a week

Health behaviors and outcomes among youth men aged 18-24 years in a rural area of north India

Survey Interview Schedule

Interview to be conducted in **private** to ensure confidentiality of the information provided.

		☐ More than once a week<br>☐ Everyday
	**Section C (Injuries and Violence)**<br>**(चोट लगने और हिंसा संबंधित)**	
**1**	During the last 12 months, how many times were you seriously injured- injury that made you miss at least one full day of usual activity?<br>पिछले बारह महीने में आप कितनी बार गंभीर रूप से घायल हुये –जिसके कारण आप अपने रोजमर्रा के काम, कम से कम एक दिन नहीं कर पाए हो?	
**2**	During the past year, what was the major cause of the most serious injury that happened to you? / पिछले एक साल के दौरान, आपके साथ सबसे गंभीर चोट का मुख्य कारण क्या था?	
	Not Applicable	☐ Yes ☐ No
	Work related Injury (काम करते हुए)	☐ Yes ☐ No
	Injury during playing (खेलते हुए)	☐ Yes ☐ No
	RTA (सरक दुर्घट्ना)	☐ Yes ☐ No
	Fight (लड़ाई के कारण)	☐ Yes ☐ No
	Others (Specify)…………………………….	☐ Yes ☐ No
**3.A**<br><br>**3.B**	Did you ever use a two-wheeler (motorbike, scooter, luna, etc)?<br>क्या आपने कभी भी दो पहिया (मोटर-साइकिल, स्कूटर, लूना, इत्यादि) चलायी है<br>Do you wear a helmet when you ride on a motorbike or scooter?<br>क्या आप मोटर-साईकिल या स्कूटर की सवारी के दौरान हेलमेट पहनते हैं?	☐ Yes ☐ No<br><br>☐ Never<br>☐ Rarely<br>☐ Sometimes<br>☐ Most of times<br>☐ Always<br>☐ Not Applicable
**4.A**<br><br>**4.B**	Did you ever use a four-wheeler (car)?<br>क्या आपने कभी भी चार पहिया (कार) चलायी है<br>Do you wear seatbelt when you ride in the car?<br>क्या आप कार में सवारी करते समय सीट बेल्ट पहनते हैं।	☐ Yes ☐ No<br><br>☐ Never<br>☐ Rarely<br>☐ Sometimes<br>☐ Most of times<br>☐ Always<br>☐ Not Applicable
**5**	During the past 30 days, how many times did you ride in a car or other vehicle driven by someone who had been drinking alcohol?<br>पिछले 30 दिनों में आपने कितनी बार कार या अन्य वाहन मे शराब पीये हुए व्यक्ति के साथ सवारी की?	___ ___ ___<br>☐ Not Applicable
**6**	During the past 30 days, how many times did you ride in a car or other vehicle when you had been drinking alcohol?<br>पिछले 30 दिनों में, आपने कितने बार शराब पिके गाड़ी चलायी?	___ ___ ___<br>☐ Not Applicable
**7**	During past 30 days, on how many days did you carry a weapon such as knife, etc.?<br>पिछले 30 दिनों में से कितने दिन आप चाकू या कोई और हथियार अपने साथ में रखा था ?	___ ___ ___
**8**	During the past 30 days, has someone threatened or injured you with a weapon such as knife, etc?<br>पिछले 30 दिनों में क्या आपको किसी ने चाकू या कोई हथियार दिखा के धमकी दी या घायल किया है?	___ ___ ___
**9**	During the past 12 months, how many times were you in a physical fight?<br>पिछले 12 महीनों में आपकी किसी के साथ कितनी बार हाथापाई हुई?	___ ___ ___
**10**	During the past 12 months, how many times have you experienced someone saying something intentionally rude or insulting to you?<br>(specify the number)<br>पिछले 12 महीनों में किसी व्यक्ति ने कितनी बार आपके साथ जानबूझकर अभद्र या अपमानजनक व्यवहार किया है ?	___ ___ ___
	**Section D (Mental Health)**<br>**(मानसिक स्वास्थ्य)**	
1	In past 12 months, did you ever feel loneliness most of the time or always?<br>पिछले 12 महीनो में क्या आपने कभी भी हमेशा या ज्यादातर बार अकेलापन का अनुभव किया है?	☐ Yes<br>☐ No
2	In past 12 months, did you ever consider/thought of attempting suicide?	☐ Yes

	पिछले 12 महीनो में क्या आपने कभी भी जान से मरने की बात सोची या दिमाग में आयी हो?	☐ No
3	In past 12 months, did you ever attempt (even single time) to commit suicide?<br>पिछले 12 महीनो में क्या आपने कभी भी जान से मरने की कोशिश की है	☐ Yes<br>☐ No

**Individual ID**		
	**Section E (Sexual Behavior)**<br>**खंड-जी (यौन व्यवहार)**	
**1**	Have you ever had sexual intercourse?<br>क्या आपने कभी भी सहवास/ सम्भोग/ यौन संबंध किया है?	☐ Yes<br>☐ No<br>**If no go to Q. 8**
**2**	Have you ever engaged in premarital sexual intercourse	☐ Yes<br>☐ No
**3**	How old were you when you had sexual intercourse first time?<br>जब आपने पहली बार सहवास किया था तब आप कितने वर्ष के थे?	__ __ Yrs
**4**	During your life, with how many people have you had sexual intercourse?<br>आपने अपने पुरे जीवन के में कितने लोगों के साथ सहवास किया है?	__ __
**5**	During the past 3 months, with how many people did you have sexual intercourse?<br>पिछले 3 महीनों में, आपने कितने लोगों के साथ सहवास किया है?	__ __
**6**	Did you drink alcohol or use drugs before you had sexual intercourse last time?<br>पिछली बार सहवास करने से पहले क्या आपने शराब या नशीले पदार्थो का इस्तेमाल किया था?	☐ Yes<br>☐ No
**7**	The last time you had sexual intercourse, did you or your partner use a condom?<br>पिछली बार जब आपने सहवास किया था, तब आपने या आपके साथी ने कॉन्डोम का इस्तेमाल किया था?	☐ Yes<br>☐ No
**8**	The last time you had sexual intercourse, which one method did you or your partner use to prevent pregnancy?<br>आखिरी बार जब आपने सहवास किया था, तब आप या आपके साथी ने गर्भावस्था को रोकने के लिए किस चीज़ का इस्तेमाल किया था?	☐ Condom<br>☐ Oral pills<br>☐ Injections<br>☐ Emergency Contraceptives<br>☐ Others (specify)___________<br>☐ None
**9**	Did anyone ever touch your private body parts without your consent? (क्या कभी किसी ने आपकी मर्जी के बिना आपके प्राइवेट बॉडी पार्ट को छुआ है)	☐ Yes<br>☐ No
**10**	Did anyone ever have intercourse with you without your consent? (क्या कभी किसी ने आपके साथ बिना आपकी मर्जी के सहवास किया है)	☐ Yes<br>☐ No
